# ENHANCING EQUITY TO QUALITY CARE FOR PERSONS WITH DEMENTIA IN RURAL COMMUNITIES THROUGH IMPLEMENTATION OF AGETECH

**DOI:** 10.1093/geroni/igae098.0918

**Published:** 2024-12-31

**Authors:** Shannon Freeman, Sarah Sousa, Davina Banner-Lukaris, Kelly Skinner

**Affiliations:** University of Northern British Columbia, Prince George, British Columbia, Canada; University of Waterloo, Waterloo, Ontario, Canada; University of Northern British Columbia, Prince George, British Columbia, Canada; University of Waterloo, Waterloo, Ontario, Canada

## Abstract

Older adults living with dementia in rural and northern communities deserve equitable access to technologies that enhances quality of life and supports aging in place. To explore contextual barriers and facilitators to implementation, we conducted a process evaluation guided by the Theoretical Domains Framework in a dementia care home newly opened in a rural and northern community. Through a partnership with the Center for Technology Adoption for Aging in the North (CTAAN), health systems leaders, and community partners, multiple technologies designed to support persons who are aging (AgeTech) were purposefully implemented to enhance clients’ care and quality of life. AgeTech included a hydroponic gardening wall, circadian lighting, and a virtual exercise program. Semi-structured interviews were held with facility staff, health systems leaders, representatives from the AgeTech companies, and implementation leads and a secondary analysis of existing documentation was conducted. Barriers to AgeTech implementation included geographic context, complexity of dementia symptoms, and limited experiences by older adults with technology. Facilitators of AgeTech included collaborative partnerships with AgeTech companies, client interest and motivation, and creation of AgeTech educational resources. Results provide insights to inform planning and policy decisions for rural AgeTech implementation initiatives, highlight considerations for ongoing AgeTech innovation and describe the engagement of community partners in the process of integrating aging technologies. Persons living with dementia can greatly benefit from use of AgeTech to support their health and wellbeing. Successful and sustainable implementation of AgeTech is possible when the AgeTech enables, empowers, and engages persons to age well.

